# A Standardized Platform for QLED Fabrication and Characterization

**DOI:** 10.1021/acs.chemmater.6c01124

**Published:** 2026-07-29

**Authors:** Yiman Xu, Grant J. Dixon, Fangyuan Jiang, Haoyu Wang, Brandi M. Cossairt, David S. Ginger, Elsa Reichmanis

**Affiliations:** † Department of Chemical and Biomolecular Engineering, 1687 Lehigh University, 124 E. Morton Street, Bethlehem, Pennsylvania 18015, United States; ‡ Department of Chemistry, 7284 University of Washington, Seattle, Washington 98195, United States

## Abstract

In recent years, quantum dot light-emitting diodes (QLEDs) have achieved record-high performance, driven by rapid advances in active materials and device engineering. However, fabrication remains a significant barrier for new researchers entering the field, and variability in measurement techniques complicates direct comparison of device performance. Here, we establish a standardized and reproducible protocol for QLED fabrication and characterization, designed to lower the technical threshold, improve experimental reliability, and ensure accurate performance assessment. The protocol was independently validated by researchers with different levels of experience. In addition, we provide detailed troubleshooting guidelines and identify key sources of variability in QLED devices. By integrating electron transport material synthesis, optimized thin-film processing, and standardized device characterization procedures, this protocol ensures consistent device performance and promotes broader adoption of QLED technology across the research community.

## Graphical abstract

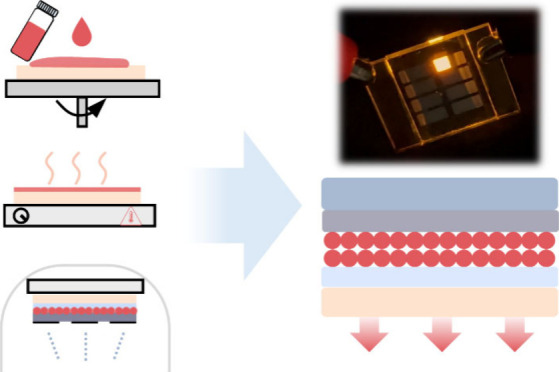

## 1 Introduction

Quantum dots (QDs) are semiconducting nanoparticles with size-dependent emission, high color purity, and excellent solution processability.^1,2^ Based on these properties, quantum dot light-emitting diodes (QLEDs) have emerged as solution-processed electroluminescent (EL) devices offering a wide color gamut and high efficiency. In a typical QLED, electrons and holes are injected from the electron transport layer (ETL) and the hole transport layer (HTL), respectively, subsequently recombining in the QD emissive layer to generate photons.

Driven by concerns over the toxicity and environmental impact of cadmium-based QDs, significant efforts have focused on cadmium-free QLEDs, such as InP and ZnSeTe. Unless otherwise specified, all QLEDs discussed in this study are inorganic and cadmium-free. Advances in material and device engineering have been reported, including the development of HTLs and ETLs with matched energy levels and carrier mobilities,^3–7^ surface ligand engineering to enhance carrier transport within the emissive layer,^8–11^ and microstructural design to improve light outcoupling efficiency.^12^ Combined with the progress in QD structure and synthesis, these advances have enabled state-of-the-art cadmium-free QLEDs to achieve high performance, with external quantum efficiencies (EQEs) over 23% for red InP-based devices,^13,14^ approaching 22% for green ZnSeTe-based devices,^15^ and exceeding 20% for blue ZnSe-based devices,^16^ accompanied by luminance levels reaching 10^5^ cd m^–2^. Moreover, a set of broadly adopted device architectures has emerged for different types of QDs, providing a reliable baseline for QLED fabrication.^17^

Despite these advances, QLED performance remains highly sensitive to fabrication and measurement conditions, particularly for researchers entering the field. QLED fabrication is based on multilayer solution processing, including the hole injection layer (HIL), HTL, QD thin film, and the ETL. Solution processing, however, involves complex fluid mechanics and is typically less controllable than vacuum deposition.^18,19^ The quality of each resulting film directly affects the performance of the QLEDs, as manufacturing defects or incomplete layer coverage can lead to charge leakage, nonradiative recombination, and efficiency loss.^14,20^ From the processing perspective, controlling solution properties, processing parameters, and interlayer mixing is therefore essential for producing uniform, defect-free films.^21,22^

Another challenge lies in QLED measurement, as most research groups rely on home-built setups without standardized device certification or a characterization checklist.^23^ While prior studies have improved LED measurement accuracy,^24–27^ missing experimental details and the lack of software control procedures have made these systems difficult to reproduce. Establishing a reliable measurement setup is often labor-intensive for first-time users, but it can be greatly simplified once standardized hardware configurations, system control, and analysis programming are fully disclosed. A well-defined protocol will enable straightforward repetition by subsequent users.

Herein, we present a comprehensive protocol from ETL ZnMgO nanoparticle (NP) synthesis and QLED fabrication to automated QLED device characterization. The objective of this work is to establish and validate a standardized, reproducible protocol for QLED fabrication and characterization, rather than a new material system or device architecture. In addition to consolidating procedures reported across the literature, the protocol incorporates practical processing guidance, experimental observations, troubleshooting recommendations, and safety considerations to facilitate reliable implementation by researchers with varying levels of experience. We identify and systematically address key sources of variability in QLED devices, particularly those associated with thin-film processing and interfacial quality. We adopted lab-made InP/ZnSe/ZnS QDs for demonstration.^20^ Across 65 devices, the average maximum EQE is 5.8%, and the average maximum luminance at 8 V is ∼8038 cd m^–2^. These values represent statistically representative device performance obtained using the protocol rather than optimized champion-device metrics, providing a practical baseline for reproducible QLED fabrication. The protocol was independently reproduced by three researchers with different levels of experience, demonstrating its accessibility and ease of adoption. While the absolute performance is not optimized for peak efficiency, the relatively narrow device-to-device variation shows the robustness and reproducibility of this protocol, making it a reliable reference for device fabrication. The QLED measurement system utilizes LabVIEW for hardware control and Python for automated data analysis, allowing for a consistent, reproducible assessment of QLED performance. This work establishes a standardized, user-friendly protocol to lower the technical threshold for QLED fabrication and facilitate reliable QLED measurements for the research community.

## 2 Progress in Cadmium-Free QLEDs

To construct red-green-blue (RGB) pixels for display applications, InP and ZnSeTe QDs are promising candidates for Cd-free QLEDs. InP can achieve stable red and green emission, while ZnSeTe with a wider bandgap can reach deep blue emission.^28,29^ A typical QLED device consists of an indium tin oxide (ITO) transparent electrode, organic semiconducting polymers as the HIL and HTL, a QD emissive layer, a ZnO or ZnMgO ETL, and a reflective Ag or Al top electrode. The thickness and uniformity should be optimized for each active layer for good interfacial contact, uniform carrier injection, and enhanced device performance.^30,31^ However, due to the intrinsic material limitations of popular active materials, challenges such as interfacial energy barriers, exciton quenching, and charge imbalance still exist.^32,33^ Recent studies have provided increasing insights into QLED stability, including positive aging, fatigue effects, and synaptic behaviors.^34–38^ Device degradation has been linked to mechanisms such as charge accumulation and leakage, interfacial reactions, and the high surface reactivity of ZnO-based ETLs.^39^ Therefore, engineering the active layers through device structure optimization is essential for enhancing carrier recombination efficiency and improving stability. Furthermore, machine learning has been applied for QLED device performance optimization and acquisition of mechanistic insights through analysis of large experimental data sets and process-performance relationships.^40,41^ Before discussing our test structure below, we briefly review current progress in the field for context.

### 2.1 ETL Engineering

Regulating energy levels and carrier mobility of the ETL/QD interface is critical to suppress exciton quenching and achieve charge-balanced QLEDs. On the ETL side, this is usually achieved by developing effective ligand passivation strategies for ZnO/ZnMgO NPs or by introducing alternative ETL materials with improved interfacial compatibility. ZnO/ZnMgO NPs are popular ETLs due to their high electron mobility, optical transparency, and solution processability.^42^ However, their intrinsic oxygen vacancies lead to electron trapping and exciton quenching at the QD/ETL interface. Various interfacial engineering strategies have been developed to overcome this issue. One strategy involves chemical passivation of ZnMgO using ligands or ions to regulate the QD/ETL interface.^4^ For instance, the strong binding affinity of F^–^ effectively passivates oxygen vacancy sites and suppresses ion migration under electrical injection, thereby reducing defect density and mitigating nonradiative recombination at the interface.^43^ Introducing an insulating interlayer between the QD layer and ZnMgO can passivate interface defects and balance the carrier in QLEDs. Wang et al.^44^ introduced a ∼5 nm polyvinylpyrrolidone (PVP) layer between InP/ZnSe/ZnS QDs and the ZnMgO layer to block excess electron injection, reduce current leakage, and passivate interface defects. The PVP-modified red QLED device showed a peak EQE of 23.5%, and T_95_ lifetime (the time required for the luminance to decrease to 95% of its initial luminance) exhibited an 11-fold improvement to over 800 h at an initial luminance of 1000 cd m^–2^. However, fabricating an ultrathin and uniform insulating layer remains challenging. Beyond the commonly used ZnO/ZnMgO NPs, Wang et al.^200^ developed a new ETL, CNT2T (3′,3′′′,3′′′′′-(1,3,5-triazine-2,4,6-triyl)­tris­(([1,1′-biphenyl]-3-carbonitrile) with sufficient electron mobility, to passivate InP/ZnSe/ZnS QD surface traps. As a result, red QLEDs fabricated with the CNT2T ETL exhibited somewhat higher EQE than ZnMgO ETL-based devices, along with a 1.4-fold improvement in EQE after aging and a longer lifetime.

### 2.2 HIL and HTL Engineering

On the HIL and HTL side, optimizing energy level alignment with the QD valence band, enhancing hole mobility, and blocking electrons can facilitate efficient hole injection, reduce QD charging, and increase device stability.

The HIL facilitates efficient hole transport from the electrode to the HTL and smooths the electrode surface, leading to improved morphology of the subsequent layers. Poly­(3,4-ethylenedioxythiophene):poly­(styrenesulfonate) (PEDOT:PSS), one of the most widely used HILs, is acidic and hygroscopic, leading to degradation of the ITO electrode and compromising device performance.^45^ Thus, an alternative inorganic nickel oxide-based HIL has been developed due to its high stability, wide bandgap, reasonable hole mobility, solution processability, and optical transparency.^46^ However, nickel oxide NPs typically exhibit poor energy level alignment with InP-based QDs. To address this, Lee et al.^47^ modified nickel oxide NPs with self-assembled molecules having intrinsic dipoles to tune their work function. They found that incorporation of a trifluoromethyl group into the molecular structure activates hole injection into InP/ZnSe/ZnS QDs without luminescence quenching. The red QLED devices exhibited an EQE of 18.8% and improved operating stability compared to QLEDs fabricated with PEDOT:PSS as the HIL. Alternatively, Wan et al.^48^ developed Li-doping and oxygen-enriching strategies to control the defects in nickel oxide NPs, lowering the valence band maximum and increasing the acceptor density. The EQE for red InP-based QLEDs increased from 19.2% to 19.9% for the modified NP HIL compared to unmodified nickel oxide NPs.

An effective HTL for QLEDs should provide efficient hole injection and transport, effective electron blocking, and robust electrochemical stability. Using a double HTL can obtain carrier balance and charge overflow. In one example, Luo et al.^49^ constructed a poly­(9,9-dioctylfluorene-*alt*-N-(4-*s*-butylphenyl)-diphenylamine) (TFB) and 2,7-dioctyl[1]­benzothieno­[3,2-*b*]­[1]­benzothiophene (C8-BTBT) bilayer. The resulting blue ZnSe-based QLED device achieved an EQE of 7.23% with the double HTL, which is ∼150% higher than that of devices based on a single HTL material. The use of a double-HTL structure, however, increases fabrication complexity and requires careful control to avoid interlayer intermixing. As an alternative, *p*-doping the HTL can lead to improved energy level alignment at the HTL/QD interface and enhance the conductivity of the HTL. As an example of this strategy, Wang et al.^50^ incorporated silver bis­(trifluoromethylsulfonyl)­imide (Ag-TFSI) as *p*-dopant into poly­(9-vinylcarbazole) (PVK) as the HTL and achieved blue ZnTeSe-based QLEDs with a peak EQE of 13.9% and a 2-fold improvement in operational lifetime. Although less studied for QLED applications, phosphonic acid-based interface modifiers have been widely used in organic light-emitting diodes,^51^ and perovskite solar cells,^52^ and have recently been demonstrated in extremely high brightness (>300 000 cd/m^2^) perovskite-based QLEDs.^53^

### 2.3 QD Surface Modification

QD surface modification provides an effective route to tailor interfacial interactions and carrier transfer at the QD/HTL and QD/ETL interface, thereby improving QLED efficiency and stability. Ultimately, good ligands need to preserve photoluminescence quantum yield (PLQY), enable facile and balanced charge injection into the QDs, and preserve good processability and film-forming properties.^54^

Ligands can maintain the colloidal stability and passivate surface defects on QDs, while simultaneously modulating their energy levels to improve energetic alignment in QLEDs.^55–57^ Efficient surface passivation and improved conductivity can be achieved by replacing the original long aliphatic ligands with short, strongly binding ligands through ligand exchange.^54,58^ For instance, Wang et al.^59^ employed a 2-aminoethanethiol (CTA) for post-treatment of QD films to passivate surface defects and nonradiative sites. CTA can also introduce interface dipoles at HTL/QD and QD/ETL interfaces, thereby elevating QD energy levels. As a result, effective surface passivation and balanced electron–hole injection enable red-QLEDs with a peak EQE of 21.2% and a T_95_ lifetime exceeding 1200 h at an initial luminance of 1000 cd m^–2^. Ligand engineering can also enable the realization of stable and functional QLED devices with flexible or pixelated architectures. Cao et al.^60^ employed a short-chain bidentate chelating ligand with high binding strength to the QD surface to construct flexible cadmium-free QLEDs. The short-chain ligand can efficiently mitigate ligand detachment during bending and inhibit nonradiative recombination. The resulting flexible devices based on blue ZnSeTe/ZnSe/ZnS QDs and green/red InP/ZnSe/ZnS QDs demonstrated a peak EQE of above 15% and high stability under bending cycles. Hahm et al.^61^ applied dual ligands on InP-based QDs, including photo-cross-linkable and dispersing ligands, to realize direct patterning up to 15 000 ppi and a passive-matrix-driven RGB QD-LED array.

In addition to ligand engineering, homogeneous blending of QDs with semiconducting molecules has been explored as a facile strategy to regulate carrier transport and mitigate structural degradation. Han et al.^62^ blended InP/ZnSe/ZnS QDs and *N*,*N*′-bis­(3-methylphenyl)-*N*,*N*′-bis­(phenyl)-9,9-dioctylfluorene (DOFL-TPD), which has good hole mobility and electron-blocking ability. The resulting film shows no observable phase separation, enabling efficient energy transfer from DOFL-TPD to the QDs. The red QLED fabricated using the blended structure exhibited an improved performance, with an EQE of 18.6% and a T_70_ lifetime (the time required for the luminance to decrease to 70% of its initial luminance) of 107772 h at 100 cd m^–2^. In blending systems, careful molecular design is required to suppress phase separation. However, blending systems generally offer less interfacial control than ligand-based approaches using chemically bonded ligands.

As the above summary demonstrates, significant progress has been made in the development of cadmium-free QLEDs through extensive efforts in surface defect passivation, energy level alignment, and charge balance optimization at the device engineering level. While innovations continue to push toward higher device efficiency and stability, establishing a standardized QLED fabrication and characterization protocol remains fundamental for ensuring reproducibility and driving future advances.

## 3 QLEDs as an Approach to QD Characterization

In photoluminescence (PL), electrons and holes are generated as pairs inside the QDs upon excitation by high-energy photons and subsequently recombine to emit light. In EL, the electrons and holes are injected from the electrodes, transported through the transport layers, and recombine within the QD layer to form photons when the device is under an applied electric field. EL can therefore be affected not only by the QD properties but also by the design of the QLED structure. In the QDs, wide-bandgap shells can protect the QDs from oxidation and degradation, suppress nonradiative recombination, and thereby enable high PLQY and stability.^63–65^ Surface ligands can effectively passivate the surface defects, minimize the trap states, and improve the PLQY of QDs.^66,67^ However, high QD PLQY does not necessarily translate to high efficiency in QLED devices.^54,68^ Several factors contribute to this discrepancy. First, the insulating nature of thick shells and long aliphatic ligands suppresses the electronic coupling between neighboring QDs, forcing charge transport between QDs to rely on hopping rather than band-like transport.^69–71^ As a result, carrier injection efficiency in QLEDs is governed by both the QD layer thickness and the shell and ligand engineering of the QDs. Second, the electric-field-induced quantum-confined Stark effect can separate the electron and hole wave functions in QDs, reducing their overlap and decreasing the exciton emission rate, leading to reduced QLED efficiency.^72–74^ Third, Joule heating, Auger recombination, and leakage currents at high current densities lead to efficiency roll-off and device degradation.^75–77^

By probing device-level responses such as carrier transport, electric-field induced effects, and efficiency roll-off, QLEDs provide a powerful operando platform for characterizing carrier transport, exciton dynamics, and stability of QDs. Spectroscopic techniques have been widely used for probing the device-level photophysics. Common measurements, such as static and transient PL spectroscopy of QLED stacks, can provide insights into radiative recombination efficiency and exciton dynamics under photon excitation.^78–80^ Advanced optoelectrical techniques, such as transient absorption, transient electroluminescence, and electro-absorption measurements under electrical driving, have been widely used to elucidate carrier dynamics in QLEDs, including nonradiative recombination, charge accumulation and leakage, and carrier trapping and detrapping.^81–85^ These measurements mainly provide fundamental insights into isolated photophysical processes, rather than capturing the collective device-level behavior under electrical operation. Therefore, a standardized QLED characterization platform remains indispensable for accurately evaluating photon generation efficiency under actual device operation conditions.

## 4 A Standardized QLED Materials Platform

Building on the above discussion of materials and device engineering strategies, we now present a detailed, standardized protocol for QLED fabrication and characterization. The reference device architecture employed in this study is ITO/PEDOT:PSS/TFB/QD/ZnMgO/Ag. This reference device was chosen because it relies on straightforward processing steps and readily accessible materials, providing a practical and reliable starting point for QLED fabrication. It also serves as a useful benchmark for troubleshooting early stage device fabrication before further optimization toward higher performance. This section describes the chemicals and materials used, details the synthesis procedure and basic characterization of ZnMgO nanoparticles, and highlights key considerations for ensuring reproducibility. In addition, practical guidance on the handling and storage of sensitive materials is provided to facilitate consistent device performance across different users and laboratories.

### 4.1 Chemicals

Zn­(Ac)_2_·2H_2_O (99.999% trace metals basis), Mg­(Ac)_2_·4H_2_O (≥98%), ethanolamine (99.0%), ethanol (≥99.5%, 200 proof), ethanol (≥99.5%, anhydrous, 200 proof), propanol (≥99.5%), dimethyl sulfoxide (DMSO) (≥99.9%), octane (≥99%), *o*-xylene (97%), ethyl acetate (≥99.5%), and Hellmanex III were purchased from Sigma–Aldrich. Tetramethylammonium hydroxide (TMAH) (98%) was purchased from Thomas Scientific. PEDOT:PSS (AI 4083) was purchased from Heraeus Epurio. TFB (M_W_ ≈ 100 000) was purchased from Montreal Optoelectronics, Inc. Encapsulation epoxy (E132) was purchased from Ossila. InP/ZnSe/ZnS QDs were synthesized as previously reported.^20^ All materials were used as received without any further purification.

*Please note that ethanolamine should be stored under a nitrogen atmosphere to prevent reaction with CO*_2_ *and O*_2_ *from air and to minimize degradation*. *PEDOT:PSS (AI 4083) should be stored in a sealed container at 5–10 °C and allowed to equilibrate to room temperature prior to use*. *The ZnMgO NPs, QD suspensions, and TFB powder should be stored in sealed amber vials in a nitrogen glovebox to protect them from oxygen, moisture, and light. According to the supplier specification, the recommended retest period for TFB is 3 years under appropriate storage conditions*.

### 4.2 InP/ZnSe/ZnS QD Synthesis

InP QD seeds are synthesized according to the literature with minor modifications, and the seeds are grown to the desired size by additional injections of tris­(trimethylsilyl)­phosphine and indium stearate.^20,86^ After surface oxide removal with hydrogen fluoride (HF), a thick ZnSe shell, followed by a thin ZnS shell is then installed on the InP cores via the strategy laid out by Choi et al., with minor modifications.^87^ The QDs are purified by three precipitation/redissolution cycles from ethanol and toluene, respectively, followed by passage through a 0.2 μm polytetrafluoroethylene (PTFE) filter. The solutions are concentrated to a dry powder under reduced pressure and resuspended in anhydrous octane at a concentration of 15 mg mL^–1^. The resultant core/shell InP/ZnSe/ZnS QDs capped with oleate ligands show strong high energy absorbance from the epitaxial zinc chalcogenide shell, as well as band edge luminescence at 604 nm with an absolute quantum yield of 93% and full width half max (FWHM) of 48 nm (Figure 1a). Time-resolved photoluminescence (TRPL) of the band edge emission shows a biexponential PL decay that was void of a fast component, indicating reduced surface/interfacial carrier trapping in the InP cores (Figure 1b). Transmission electron microscopy (TEM) reveals an ensemble of pseudospherical particle morphologies, confirming epitaxial shell growth with minimal lobe formation in the zinc chalcogenide shell (Figure 1c).

**Figure 1:**
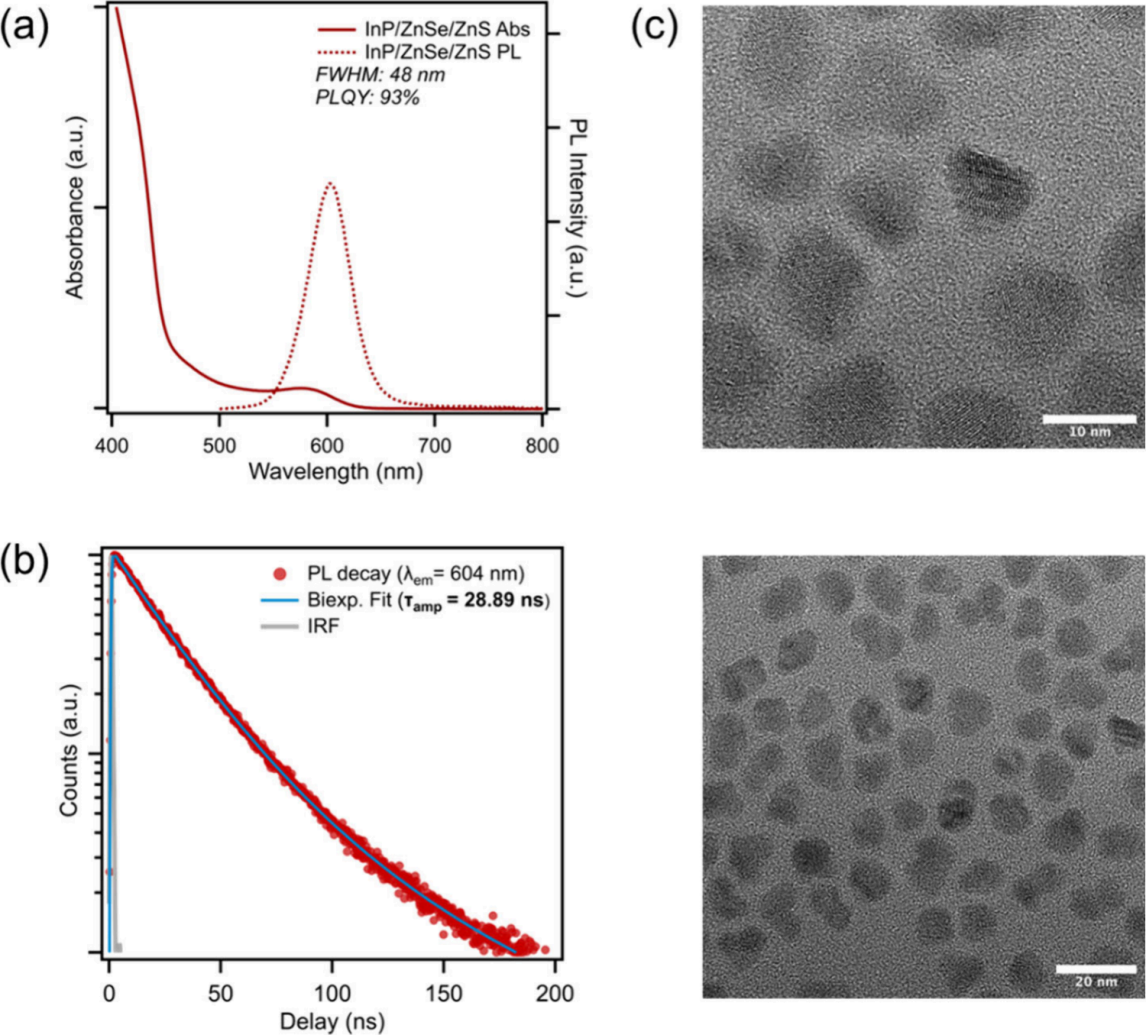
InP/ZnSe/ZnS QD characterization: (a) absorbance, photoluminescence, and PLQY; (b) time-resolved PL decay fit to a biexponential function; and (c) TEM micrographs showing pseudospherical morphology.

### 4.3 Materials

Patterned ITO glass substrates (S211), substrate rack (E101), annealing and cleaning beaker (C191), and deposition mask (E501-SPC) were purchased from Ossila. Poly­(ether sulfone) (PES) filters (0.45 μm) and PTFE filters (0.22 μm) were purchased from Denville Scientific.

### 4.4 Zn_0.85_Mg_0.15_O Nanoparticle Synthesis

*Please note that this protocol can also be used for ZnO NPs and Zn_0.9_Mg_0.1_O NP synthesis by changing the ratio of Zn and Mg precursors.*

The Zn_0.85_Mg_0.15_O NPs are prepared according to the method reported with some modifications.^88,89^ TMAH (5 mmol) in ethanol (10 mL) is added dropwise, using an addition funnel, at a rate of ∼8 mL min^–1^ to a 100 mL round-bottom flask containing Zn­(Ac)_2_·2H_2_O (2.55 mmol) and Mg­(Ac)_2_·4H_2_O (0.45 mmol) dissolved in DMSO (30 mL) under stirring at 700 rpm (Figure 2a). After the addition of TMAH, the solution is stirred for 1 h in ambient conditions. The product is divided equally into three 50 mL centrifuge tubes and precipitated by adding ethyl acetate (∼30 mL to each tube) until a white cloud appeared, indicating NP precipitation (Figure 2b). The mixture is then centrifuged at 8000 rpm for 5 min at 25 °C. After centrifugation, the NPs are fully precipitated, yielding a clear supernatant (Figure 2c). The supernatant is discarded, and the NPs are redispersed in anhydrous ethanol. It should be noted that exposure to water can induce surface site reconfiguration, resulting in difficulties in redispersing the dried powder.^90^ Brief sonication for 1 min can facilitate NP redispersion. Ethanolamine (120 μL) is added to stabilize the NPs, and the solution is stirred for 1 h to ensure effective ligand binding on the NP surfaces. The NPs are washed a second time with anhydrous ethyl acetate by centrifuging at 8000 rpm for 5 min at 25 °C. The supernatant is discarded. To maximize antisolvent removal, the NPs are further blow-dried using N_2_. The final product is redispersed in anhydrous ethanol (∼6 mL) to a concentration of ∼30 mg/mL. The suspension is centrifuged at 8000 rpm for 1 min to separate aggregated NPs from the fully dispersed particles in the supernatant. The supernatant is then transferred to vials and transferred to a N_2_ glovebox for use (Figure 2d).

**Figure 2:**
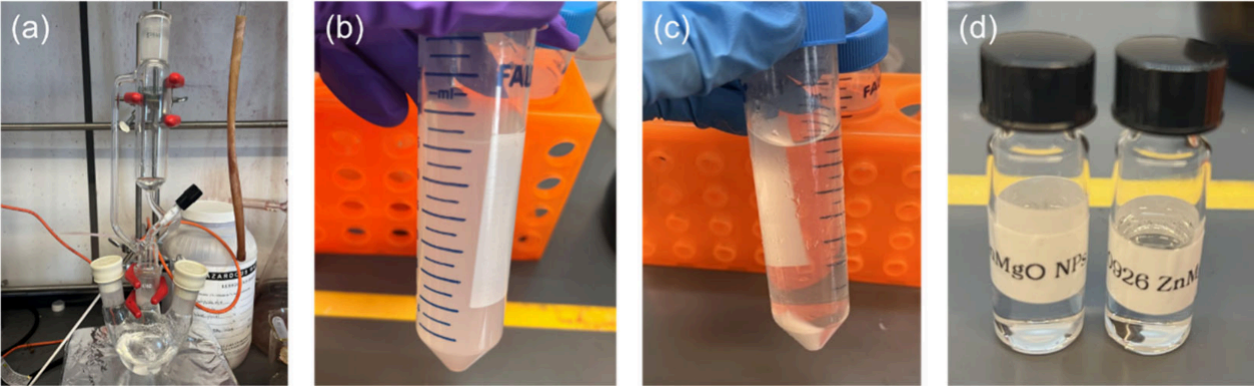
(a) Experimental setup for Zn_0.85_Mg_0.15_O NPs synthesis. (b) Formation of a white colloidal suspension upon addition of the antisolvent. (c) The NPs fully precipitated after centrifugation. (d) The resulting NP samples.

*Warning: TMAH is highly toxic and can cause severe skin burns. Handle with extreme caution and always use appropriate personal protective equipment (PPE).*

NP morphology was evaluated using TEM, where highly crystalline ZnMgO NPs with a diameter of around 5 nm are demonstrated (Figure 3a). From the UV–vis spectra, the Tauc plot is used to evaluate the ZnMgO energy bandgap, which is determined to be 3.55 eV (Figure 3b).

**Figure 3:**
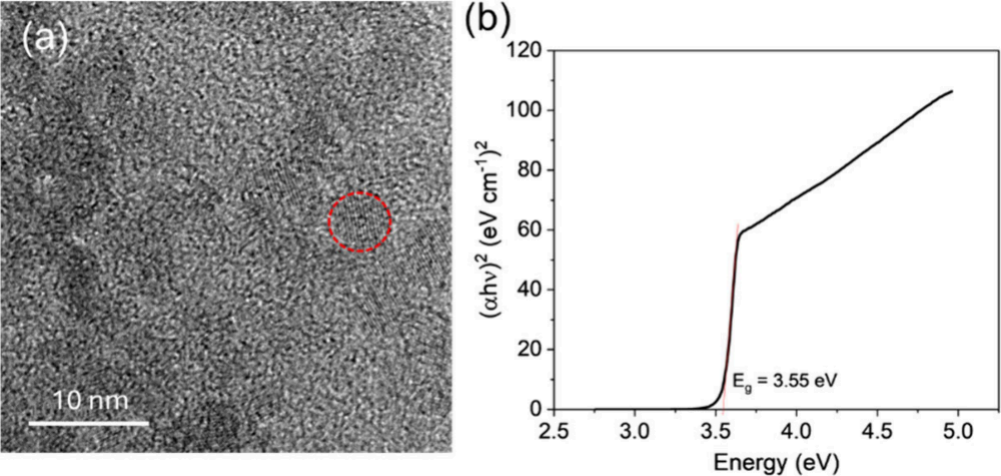
(a) TEM image showing ZnMgO NP morphology. The red circle highlights a single NP. (b) Tauc plot derived from the UV–vis absorption spectrum of the ZnMgO NP suspension.

## 5 QLED Fabrication

This section discusses the step-by-step fabrication procedures of QLEDs with device structure of ITO/PEDOT:PSS/TFB/QD/ZnMgO/Ag. The device fabrication process is shown in ). We provide guidance for troubleshooting common sources of inconsistency.

### 5.1 Substrate Preparation

ITO-coated glass substrates (sheet resistance ∼20 Ω sq^–1^) are loaded into a substrate rack and placed in an annealing and cleaning beaker containing an aqueous Hellmanex III solution (∼2 vol % in deionized water). The substrates are then immersed in a warm water bath and sonicated in a bath sonicator for 15 min, followed by thorough rinsing with deionized water (Figure 4a). They are then immersed in isopropanol alcohol (IPA), placed in a warm water bath, and sonicated for 15 min. The substrates are then thoroughly dried under a stream of dry N_2_ and treated with air plasma (18 W) for 10 min to remove organic contamination and increase substrate hydrophilicity (Figure 4b,c). If the ITO substrates will not be used immediately after cleaning, contamination from dust should be minimized by either immersing them in IPA or sealing them in a clean container. For the procedures described here, the ITO substrates are used immediately following plasma treatment to prevent loss of surface hydrophilicity.

**Figure 4:**
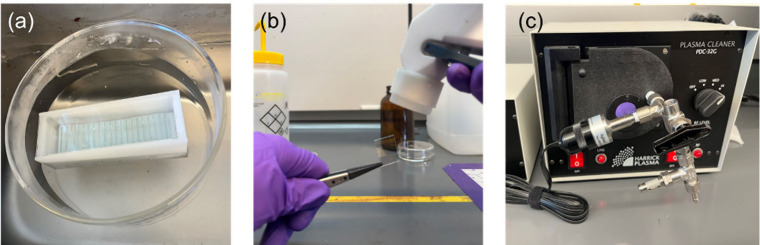
Photographs showing (a) substrates washed in solvent using a bath sonicator, (b) substrates blow-dried with a nitrogen gun, and (c) substrates treated with air plasma.

### 5.2 QLED Active Layer Deposition

Each active layer is sequentially coated via spin coating using the parameters provided in Table 1. PEDOT:PSS was deposited via static spin coating to ensure complete wetting and uniform coverage on the ITO electrode. In contrast, subsequent layers were deposited using dynamic spin coating to minimize solvent-induced intermixing and achieve better thickness control. Note that in each case, care should be taken to center the substrate on the chuck to avoid damage.

**Table 1:** Table of Spin Coating Parameters

Material	Coating method	Steps	Acceleration
PEDOT:PSS	Static	Step 1: 500 rpm 5 s	Step 0–1: 500 rpm s^–1^
		Step 2: 3000 rpm 50 s	Step 1–2: 2500 rpm s^–1^
TFB	Dynamic	Step 1: 2000 rpm 30 s	–
QD	Dynamic	Step 1: 3000 rpm 30 s	–
ZnMgO	Dynamic	Step 1: 2000 rpm 30 s	–

#### 5.2.1 PEDOT:PSS HIL Deposition

First, the PEDOT:PSS (AI 4083) solution is filtered through a 0.45 μm PES filter attached to a syringe. Glass or polypropylene (PP) syringes are recommended to minimize contamination and ensure reproducibility, whereas polystyrene (PS) syringes with rubber plungers should be avoided due to potential contamination of the aqueous dispersion. Needles should not be attached to reduce the risk of accidental injury. A cleaned ITO substrate is then placed on the spin coating chuck, and sufficient filtered PEDOT:PSS solution (approximately 75 μL) is pipetted onto the substrate to cover at least two-thirds of the substrate surface. The pipet tip should be positioned close to, but not in contact with, the surface, and the solution should be dispensed slowly onto the ITO glass to avoid bubble formation and flow disturbance. After PEDOT:PSS dispense, spin coating is performed at 500 rpm for 5 s to spread the solution uniformly across the substrate, and 3000 rpm for 50 s to remove the excess solution and define the final thickness. The coated substrates are then placed on a hot plate (150 °C), annealed for 15 min in ambient air, and subsequently transferred to a N_2_ glovebox. *After PEDOT:PSS deposition, subsequent device layers are deposited in a N*_2_ *glovebox via dynamic spin coating*.

#### 5.2.2 TFB HTL Deposition

In a N_2_ glovebox, TFB (8 mg mL^–1^) is added to a vial fitted with a magnetic stir bar and containing the requisite amount of o-xylene. The resulting solution is stirred on a hot plate at 55 °C for at least 30 min to fully dissolve the polymer. The substrate is then placed onto the chuck of the spin coating apparatus, and spinning at a rate of 2000 rpm is initiated. A total of 30 μL of the TFB solution is then dispensed onto the spinning substrate. The solution is dispensed in a single step using the first stop of the pipet to minimize bubble formation and intermixing between the as-forming solidified TFB layer and remaining TFB solution. After the TFB film formation is complete, the coated substrates are placed on a hot plate and annealed at 150 °C for 30 min. An annealing temperature of 150 °C was selected as the optimal condition for QLED devices based on published reports that it provided for TFB films with good mechanical hardness and optimal hole transport properties.^91^

#### 5.2.3 InP/ZnSe/ZnS QD Deposition

In a N_2_ glovebox, an InP/ZnSe/ZnS QD octane suspension (15 mg mL^–1^) is deposited onto the ITO/HIL/HTL substrate via dynamic spin coating, whereby the QD suspension (25 μL) is pipetted onto the spinning substrate (3000 rpm). The QD-coated substrates are then placed onto a hot plate (120 °C) and annealed for 30 min.

#### 5.2.4 ZnMgO ETL Deposition

Prior to deposition of the ETL onto the ITO/HIL/HTL/QD device stack, the as-prepared ZnMgO ethanol suspension (vide supra) is filtered through a 0.22 μm PTFE filter prior to use. Next, using a micropipette, 30 μL of the ZnMgO suspension (30 mg mL^–1^) is dispensed onto a spinning ITO/HIL/HTL/QD substrate spinning at a rate of 2000 rpm. The ETL-coated substrates are then placed onto a hot plate maintained at 90 °C and annealed for 15 min.

#### 5.2.5 Electrode Deposition

The substrates with active layers deposited in the above coating steps are shown in Figure 5a–d. In the electrode design, partial overlap between the top (Ag in this case) and bottom (ITO) electrodes is required. Therefore, a small portion of the active layers is removed using either a cotton swab wetted with ethanol or a razor blade to expose the bottom electrode (Figure 5e,f). The coated substrates are then transferred to a thermal evaporator, housed in the N_2_ glovebox, for top electrode (Ag) deposition. Thermal evaporation was carried out in a high-vacuum chamber equipped with a turbomolecular pump backed by a mechanical pump. Prior to initiating the deposition step, a shadow mask (Ossila) is placed directly on the substrates in a manner that ensures proper alignment of the substrate and mask (Figure 5g). Finally, the Ag electrode (∼100 nm) is deposited in vacuum at a pressure of ∼3 × 10^–6^ mbar, at a rate of 1 Å s^–1^ using a thermal evaporator. A completed QLED device is presented in Figure 5h.

**Figure 5:**
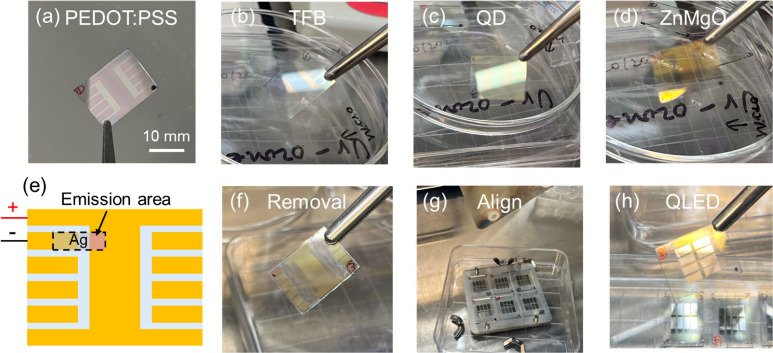
Cleaned ITO substrates sequentially spin-coated with (a) PEDOT:PSS, (b) TFB, (c) QD, and (d) ZnMgO layers. (e) Schematic illustration of the commercial patterned ITO glass with active layers and an Ag electrode, and the definition of the emission area. (f) The coated substrate with part of the active layers removed to expose the electrode. (g) Coated substrates placed into and aligned with a shadow mask prior to Ag deposition. (h) A completed QLED device.

#### 5.2.6 QLED Encapsulation

While in the N_2_ glovebox environment, the as-prepared QLED devices are subsequently encapsulated to protect them from oxygen and moisture to enable testing under ambient conditions. A single droplet of epoxy is applied to the active side of the device, after which a cover glass is placed on top to facilitate uniform spread of the epoxy (Figure 6). The cover glass is gently pressed to eliminate any trapped bubbles. The epoxy is cured under a long-wave UV lamp (365 nm) equipped with a 4 W tube and a filter assembly for approximately 15 min, as determined by the absence of visible flow or surface stickiness, indicating sufficient cross-linking for device testing.

**Figure 6:**
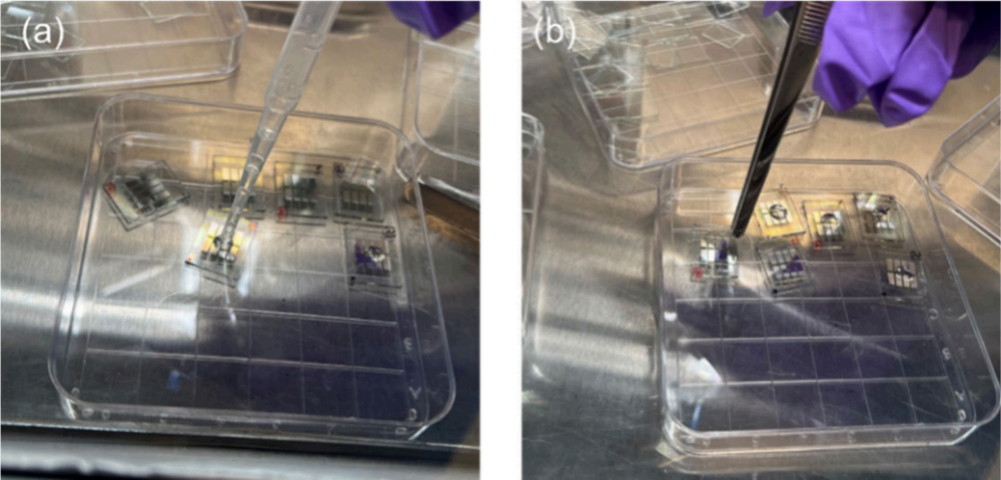
Photographs showing (a) a drop of epoxy applied to the active side of the device and (b) a cover glass placed onto the epoxy to ensure uniform spreading.

In total, 65 QLED devices were fabricated over a 3-month period following the process sequence described above. The device characteristics were then measured using a laboratory-built measurement system (vide infra). Typical performance curves for one device are presented in Figure 7a,b. The EL spectra remain sharp (full width at half-maximum of ∼50 nm) and exhibit no peak shift under different driving voltages (Figure 7c). At an initial luminance of ∼200 cd m^–2^, the T_95_ lifetime was approximately 20 min, and the luminance decay exhibited an exponential trend, which slowed after the luminance decreased to about 60% of its initial value (Figure 7d). The short lifetime indicates that the device experiences rapid degradation, which may result from QD ligand detachment and organic layer degradation.^92^ For the set of 65 devices, the average maximum EQE was 5.8% with a standard deviation of 0.4%, and the average maximum luminance at 8 V was ∼8038 cd m^–2^ with a standard deviation of 2381 cd m^–2^ (Figure 8, Operator 1). To evaluate the reproducibility and accessibility of the protocol, two more independent researchers with different levels of experience performed the QLED fabrication using the same batch of materials. One researcher was a new graduate student with no prior experience in optoelectronic device fabrication (Operator 2), while the other was a postdoctoral researcher with experience in thin-film optoelectronic device fabrication (Operator 3). Both researchers followed the protocol with minimal mentoring in our lab. Their datasets were obtained from the first several batches of devices fabricated during their initial attempts, and the resulting device performance was comparable to that of the original experiments. All measured devices were included in the statistical analysis without intentionally selecting high-performing samples, providing a transparent and realistic assessment of protocol reproducibility and fabrication robustness. Variations in performance may arise from differences between fabrication runs and/or chemical degradation, as well as from changes in the concentration and aggregation state of the QD materials over the course of a few months’ storage. Nevertheless, all three operators obtained similar performance distributions, demonstrating the reproducibility and robustness of the fabrication protocol under typical laboratory conditions. While all runs were similar, the later runs prepared with the oldest stock solutions (Operator 3) exhibited slightly lower performance, consistent with the expected effects of prolonged storage (Figure 7).

**Figure 7:**
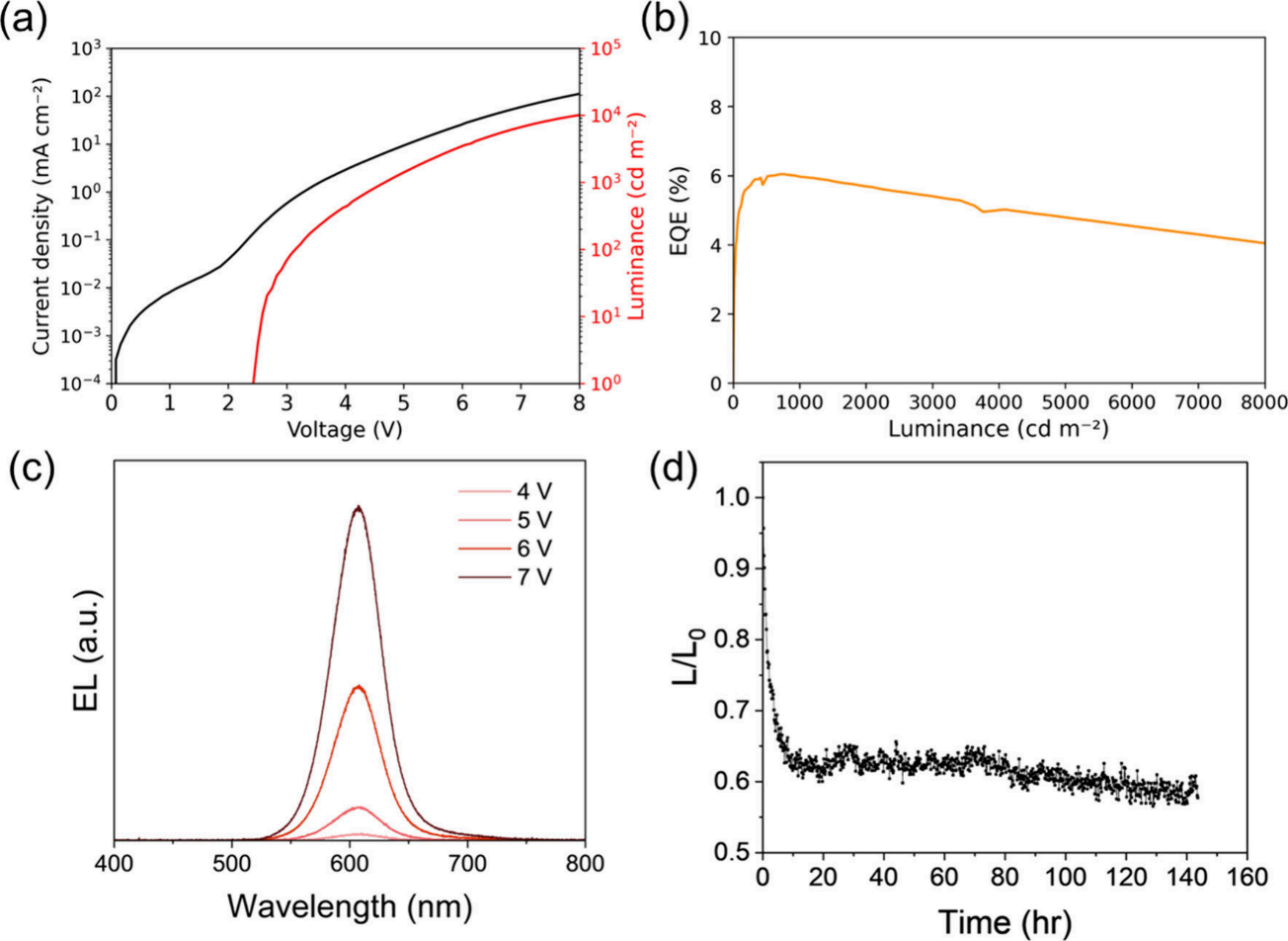
Performance of the QLED devices: (a) *J*–*V*–*L* characteristics, (b) EQE as a function of luminance, (c) EL spectra under different driving voltages, and (d) device lifetime.

**Figure 8:**
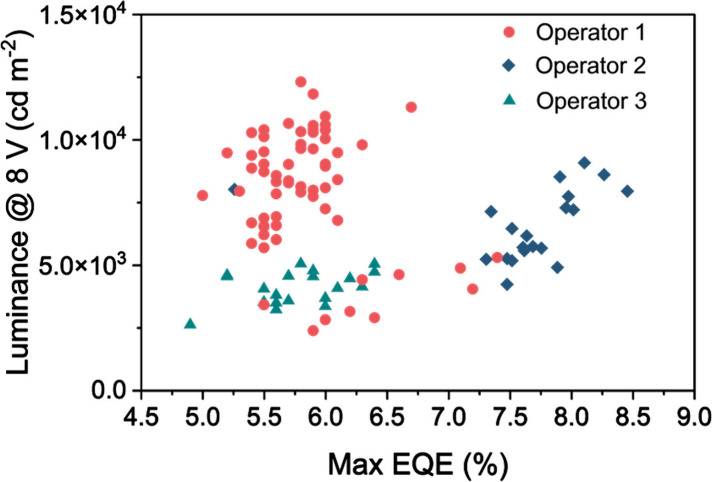
Statistical analysis of the luminance at 8 V and the maximum EQE of QLED devices fabricated by different operators between different fabrication runs.

#### 5.2.7 Troubleshooting Guide for QLED Fabrication

To help new users and improve reproducibility, common fabrication issues identified during repeated device fabrication, along with their possible causes and solutions, are given in Table 2.

**Table 2:** Common Fabrication Issues Encountered during Repeated Device Fabrication

Issue	Possible Causes and Solutions
PEDOT:PSS does not wet the ITO surface	Verify that the ITO glass is thoroughly cleaned and that the air/oxygen plasma or UV-ozone treatment time and intensity are sufficient (check wetting of a water drop/water contact angle). Ensure that the PEDOT:PSS is properly stored in a sealed container at 5–10 °C and used within its shelf life (12 months).
Defects in the active layers	Defects may originate from bubbles, impurities, or aggregates present in the active solution. The solution can be filtered or centrifuged to remove impurities and aggregates prior to use. Bubble formation should be minimized during pipetting by carefully aspirating and dispensing the solution. Substrate cleaning and storage are important. Clean, low-dust environments are beneficial.
Nonuniformity in the active layers	The solution should be dispensed in a single step to avoid nonuniform film formation caused by partial redissolution during repeated solution addition. Additionally, nonuniformity may result from vacuum-induced deformation of the substrate by the spin-coater chuck if thin substrates are used.
Inconsistent device performance	Device performance inconsistency is often related to variations in solution concentration, defects in the film, and degradation of the active materials. Solvent evaporation over time can increase the concentration of the active solution, leading to thicker films. In this case, diluting the solution with the original solvent will help. In addition, film uniformity and the presence of defects should be carefully examined. The degradation of the active materials, particularly QDs, may occur over time even when stored in a glovebox. To mitigate this issue, freshly synthesized QDs and other active materials should be used.
Current leakage and short circuit in QLEDs	Current leakage typically originates from defects in the device. If the leakage current is large, direct contact between the QD layer and the electrodes should be considered. Defects in the active layers can be identified through simple visual inspection under ambient light during film processing, while characterization techniques such as atomic force microscopy (AFM) and scanning electron microscopy (SEM) are effective for detecting smaller-scale defects. Short circuits may also occur when the active layers are too thin. In such cases, increasing the solution concentration and appropriately adjusting the coating speed should be considered.

## 6 QLED Characterization

In this section, we describe a comprehensive and standardized QLED characterization framework, including the measurement system design, photon flux determination, and evaluation of key device metrics. We also developed open-source automated data acquisition and analysis tools that enhance measurement accuracy, reduce user-dependent variability, and enable reproducible comparison of QLED performance across different studies. The complete QLED characterization procedure is demonstrated in .

### 6.1 QLED Measurement System Design and Mechanism

To calculate the EQE and luminance, the photon flux, defined as the rate at which photons pass through a given solid angle per unit time, must be measured. Three ways to measure photon flux include the following: (1) a detector whose area is much larger than that of the QLED is either placed close to the QLED, or placed sufficiently far away so that the QLED pixels can be assumed to be a point light source (Figure 9a); (2) an integrating sphere can be used to collect all the emitted photons, which are detected by a photodetector mounted on the sphere (Figure 9b); (3) a detector mounted on a goniometer can be used to determine the full angular distribution of emission (Figure 9c).^93^ In all configurations, the emission from the edges of the device should be eliminated to avoid overestimation of the forward photon flux as light emitted into substrate waveguide modes will not typically be useful.

**Figure 9:**
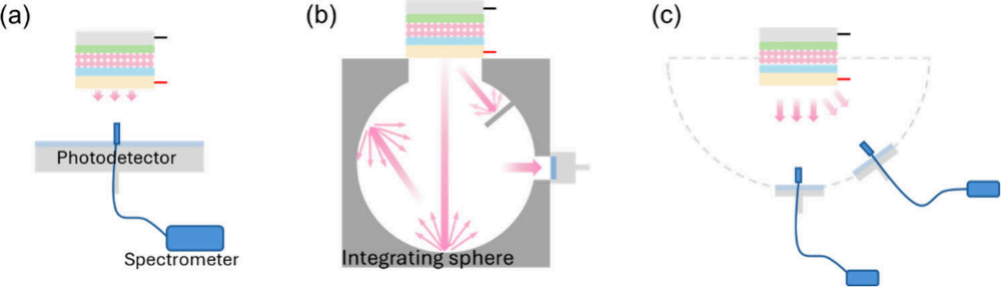
Typical apparatus for photon flux measurement of QLEDs.

QLED figures of merit include EQE, luminance, and lifetime. EQE is defined as the number of photons emitted from the front hemisphere of the device divided by the number of electrons injected into the device per unit time. Luminance is defined as the luminous intensity, weighted by the standard human eye sensitivity, per unit projected area in a given direction, with SI units of candela per square meter (cd m^–2^). The lifetime is measured by evaluating the luminance when driving the device at a constant current density. The *T*_50_ and *T*_95_ lifetimes are defined as the time required for the luminance to decay to 50% and 95% of its initial value, respectively. The measured lifetime depends on the initial luminance, with higher initial luminance resulting in a shorter lifetime.

Here, we adopted the first approach since it is relatively straightforward to realize and presents a cost-effective solution. Table 3 provides a full list of necessary components required for implementation. The supposition that a QLED pixel represents a point light source requires the added assumption that the emission is Lambertian, which means the LED device emits with equal radiance into any solid angle within the forward viewing hemisphere.^94^ It is important to verify the angular emission profile of the target devices before applying this setup. As a practical check, users may measure emission intensity and spectra at several viewing angles, such as 0°, 30°, 60°, 90°, 120°, and 150°, while keeping the detector distance fixed. The measured intensity should approximately follow the expected Lambertian angular dependence, and the spectral shape should remain largely unchanged across different angles. Significant deviation from Lambertian behavior or strong angular spectral variation would indicate that the simplified photodiode-based geometry may introduce additional measurement error. In the methodology developed here, light is collected from a fixed spot on the QLED surface at a distance substantially greater than the dimensions of the emitting area of the device (>20 times), such that the detected light subtends only a small solid angle centered on the surface normal.^93^ Specifically, a calibrated silicon photodiode serves as a photodetector and is placed perpendicular and on-axis in front of the QLEDs at a distance of 10 cm from the emitting surface (Figure 10). Given the dimensions of each QLED (2 mm × 2 mm), the detector is sufficiently far from the QLED to ensure that the subtended solid angle can be approximated as infinitesimally small. At the same time, the collected signal must remain sufficiently large to ensure accurate detection.

**Figure 10:**
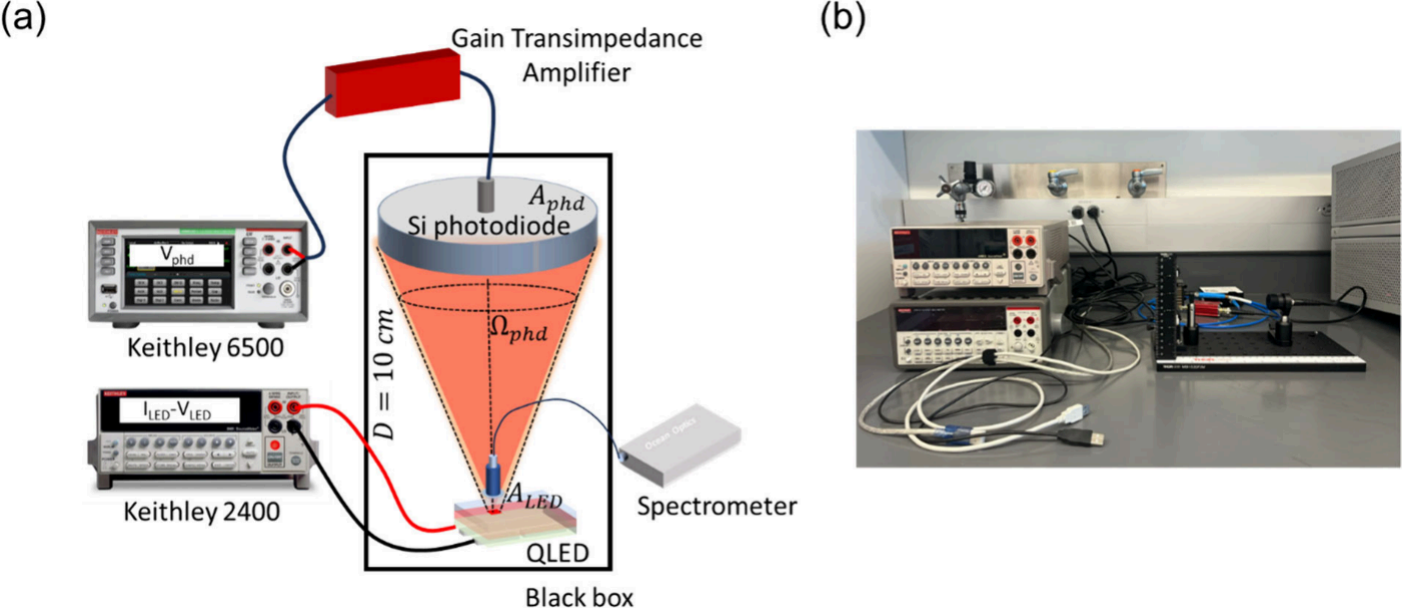
(a) Schematic illustration and (b) a photo of the QLED measurement system.

**Table 3:** Components for the QLED Measurement System

Key Component	Model	Note
Si Switchable Gain Detector	Thorlabs PDA100A2	Measure photon flux. The responsivity plot can be found in Thorlabs Web site
Multimeter	Keithley DMM6500	Connect with the photodetector and measure the photovoltage
Source Meter	Keithley 2400	Connect with the test board, supply the voltage, and measure current
Spectrometer	Ocean Optics FLAME-T-VIS-NIR	Measure the spectra of the devices
Optical Fiber	Ocean Optics P600–2-UV–vis	Connect with the spectrometer
Aluminum Breadboard	Thorlabs	Mechanical support
LED Test Board	Ossila P2008A1	Make electrical connections to the devices and control on/off
		

Accessories	Model	Note
Trigger Cable	Keithley 8503 DIN to BNC Trigger Cable	Connect the multimeter and source meter
±12 VDC Regulated Linear Power Supply	Thorlabs LDS12B	Power supply for photodetector
Ø 1/2 in. Post Holders and Optical Posts	Thorlabs	Support the photodetector and optical fiber
BNC Cables and Adapters	Thorlabs	Connect the multimeter and source meter with the photodetector and the test board
Post-Mountable Ferrule Clamps	Thorlabs	Mount the optical fiber
Plate Holders	Thorlabs FP02	Hold the LED test board

Additional considerations include the following points. The photodiode should be selected based on the emission wavelength of the QLED of interest. A transimpedance amplifier with gain is used to convert the photocurrent into a voltage signal and amplify low photocurrents to a measurable level, thereby facilitating multimeter readout and reducing measurement error. Alternatively, a Si switchable gain detector, which integrates a photodiode with a switchable gain transimpedance amplifier, can be used to simplify the measurement setup. To minimize the influence of ambient light, a black box covers the photodiode and QLED while measuring the photon flux. A multimeter collects the voltage signal from the photodiode, which is used to calculate photon flux and luminance. At the same time, a source meter supplies voltage to the QLED and reads current. The device spectrum is measured separately by positioning an optical fiber close to the device surface at a specified driving voltage. The spectra are measured by a spectrometer and collected using the accompanying software from Ocean Optics.

### 6.2 Automated Data Acquisition

To build a straightforward user interface, the LabVIEW interface is used to control both the multimeter and source meter (code available at https://github.com/YIX421/LABVIEW-QLED-Measurement). Here, the QLED current and luminance voltage must be sampled under the same driving voltage. To realize this synchronous sampling, the multimeter and source meter are connected with a trigger cable, and the multimeter is programmed to trigger the source meter at each sample point. The NI-VISA package and the drivers for the Keithley 2400 and DMM6500 are available for download from the National Instruments support webpage. These drivers enable programmatic control of the instruments.

The LabVIEW user interface, depicted in Figure 11, employs graphical coding to enable the system’s full functionality. This interface includes various settings: the file path for data storage, configuration options for the Keithley DMM6500 multimeter and 2400 source meter, and graphs that display output data, providing a quick assessment of data quality. The parameters that must be set prior to measurement are shown in blue. The maximum and minimum voltages can be set according to experimental requirements. The timeout should be set longer than a single measurement cycle. Moreover, the voltage sweep speed can be adjusted by introducing a delay at each trigger. The number of power line cycles (NPLC) of the multimeter can also be altered. A larger NPLC increases measurement resolution and accuracy but slows the measurement rate.

**Figure 11:**
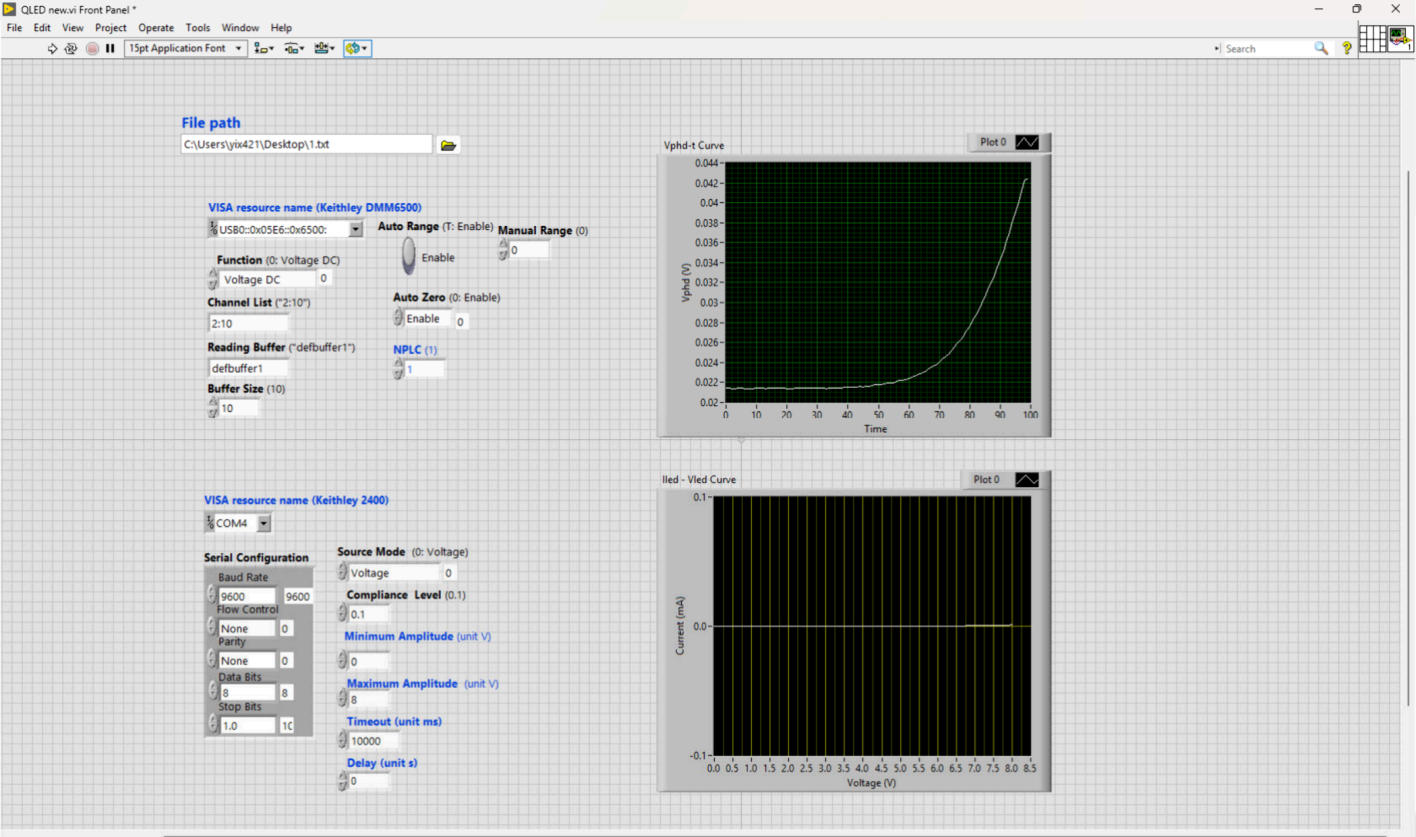
LabVIEW user interface for the QLED characterization system.

Once configured correctly, the LabVIEW system can efficiently control the entire hardware setup under the target parameters, deliver results with a single click, and save the data to text files. The spectrum of the device is measured afterward by the spectrometer. The device lifetime can be measured by the front-panel configuration of the multimeter and source meter. The source meter should be set to a constant current mode, and the multimeter measures the photovoltage constantly. To avoid storage overflow, a delay should be placed before each measurement in a trigger flow (Figure 12). After the photovoltage has dropped to a certain level, the data can be extracted by using a USB drive.

**Figure 12:**
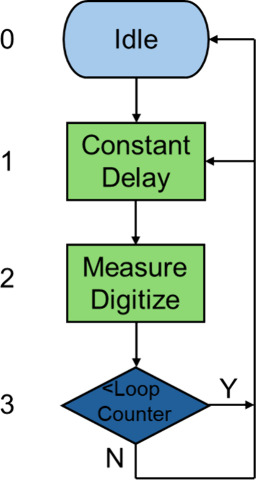
Trigger flow in DMM6500 multimeter for lifetime measurement.

### 6.3 Automated Data Analysis

To comprehensively assess QLED performance, various key metrics can be derived from the raw data gathered during data acquisition. The equations required for detailed calculations are given in Table 4.

**Table 4:** Summary of Parameter Determination in QLEDs^a^

Quantity	Symbol (unit)	Expression
Photon Flux	Φ_phd_ (photons s^–1^ sr^–1^)	$\Phi_{\mathrm{phd}}=\frac{C\cdot V_{\mathrm{phd}}\cdot \int S\left(\lambda \right)\;d\lambda }{\Omega_{\mathrm{phd}}\cdot \int Q\left(\lambda \right)\cdot S\left(\lambda \right)\;d\lambda }$
External Quantum Efficiency	EQE	$\mathrm{EQE}=\frac{\pi \cdot \Phi_{\mathrm{phd}}}{I_{\mathrm{LED}}/q}$
Luminance	*L* (cd m^–2^)	$L=\frac{K_{m}\cdot \Phi_{\mathrm{phd}}\cdot \int V\left(\lambda \right)\cdot S\left(\lambda \right)\cdot \frac{hc}{\lambda }\;d\lambda }{A_{\mathrm{LED}}\cdot \int S\left(\lambda \right)\;d\lambda }$
Current Density	J (mA cm^–2^)	$J=\frac{I_{\mathrm{LED}}}{A_{\mathrm{LED}}}$

a Definition of terms: *C* is conversion factor between the output voltage of the transimpedance amplifier and the electron flux, *S*(λ) is normalized EL spectra of QLEDs, *Q*(λ) is photodiode quantum efficiency, *V*(λ) is photopic factor, Ω_phd_ is the solid angle, *q* is elementary charge, *V*_phd_ is voltage of photodetector measured by the multimeter, *K*_m_ is a constant corresponding to the maximum photopic luminous efficacy (683 lm W^–1^) defined by the CIE for photopic vision, *A*_LED_ is the active area of QLED, and *I*_LED_ is the current flow through the QLED measured by the source meter, *h* is Planck’s constant, *c* is the speed of light in vacuum, and λ is the wavelength of the emitted light.

The EQE is defined as

$$EQE=\frac{N_{\mathrm{photon}}}{N_{\mathrm{electron}}}$$

where *N*_photon_ is the number of photons emitted into the forward hemisphere of the device per unit time and *N*_electron_ is the number of electrons injected into the device per unit time. To measure *N*_photon_, the EL spectrum is collected by the spectrometer, and the absolute emission intensity is determined using the photodiode. In this case, *N*_photon_ can be calculated based on the Lambertian assumption by using

$$N_{\mathrm{photon}}=\int_{0}^{2\pi }\int_{0}^{\pi /2}\Phi_{\mathrm{phd}}\cdot \cos\theta \cdot \sin\theta \;d\theta \;d\phi =\pi {\cdot \Phi }_{\mathrm{phd}}$$

where Φ_phd_ (photons s^–1^ sr^–1^) is the photon intensity per unit solid angle along the surface-normal direction (assuming the direction-independent Lambertian assumption). The responsivity of the photodetector is an essential factor that determines the photon flux intensity at different wavelengths and can be found on the manufacturer’s Web site. The responsivity, *R* (λ) (A/W), in the plot can be translated to photodiode quantum efficiency *Q*(λ) by using

$$Q\left(\lambda \right)=\frac{R\left(\lambda \right)hc}{q\lambda }$$

where *h* is Planck’s constant (6.626 × 10^–34^ J·s), *c* is the speed of light in vacuum (3 × 10^–8^ m·s^–1^), *q* is the elementary charge (1.602 × 10^–19^ C), and λ is the wavelength (m). Since the EL spectrum is broadband, photons at different wavelengths generate different photocurrents in the photodiode due to the wavelength-dependent responsivity. Therefore, an effective quantum efficiency ⟨*Q*⟩ is introduced to represent the average probability of charge generation per photon across the emission spectrum. The effective quantum efficiency is defined as

$$\left\langle Q\right\rangle =\frac{\int Q\left(\lambda \right)\cdot S\left(\lambda \right)\;d\lambda }{\int S\left(\lambda \right)\;d\lambda }$$

where *S*(λ) is the normalized EL spectra of QLEDs. To convert the measured photovoltage to electron flux, the conversion factor, *C* (electrons s^–1^ V^–1^), is represented by

$$C={\left(qR_{\mathrm{TIA}}\right)}^{-1}$$

where *R*_TIA_ (Ω) is the transimpedance gain. As noted above, under these geometric conditions, each QLED pixel can be approximated as a point source.

Based on the stated requirements, the QLED pixel can be taken as a point emitter (Figure 13). The circular photodetector aperture subtends a conical solid angle at the emission point, with a half angle θ. The solid angle, Ω_phd_ (sr), can be calculated by the equation

$$\Omega_{\mathrm{phd}}=2\pi \left(1-\cos\theta \right)=2\pi \left(1-\frac{D}{\sqrt{D^{2}+R^{2}}}\right)$$

where *R* (m) is the radius of the photodetector and *D* (m) is the distance between the photodetector and the device. The photon flux can be calculated by

$$\Phi_{\mathrm{phd}}=\frac{C\cdot V_{\mathrm{phd}}}{\Omega_{\mathrm{phd}}\cdot \left\langle Q\right\rangle }=\frac{C\cdot V_{\mathrm{phd}}\cdot \int S\left(\lambda \right)\;d\lambda }{\Omega_{\mathrm{phd}}\cdot \int Q\left(\lambda \right)\cdot S\left(\lambda \right)\;d\lambda }$$

where *V*_phd_ (V) is the photovoltage from the gain photodetector. To account for the baseline response of the photodetector, a background voltage can be recorded under dark conditions and subtracted from the measured signal during data processing. Therefore, the EQE can be calculated by

$$\mathrm{EQE}=\frac{\pi {\cdot \Phi }_{\mathrm{phd}}}{I_{\mathrm{LED}}/q}$$

where Φ_phd_ is the photon flux measured by the photodetector along the surface normal, and the factor of π accounts for conversion to total hemispherical emission under the Lambertian assumption. *I*_LED_ (A) is the current flow through the QLED.

**Figure 13:**
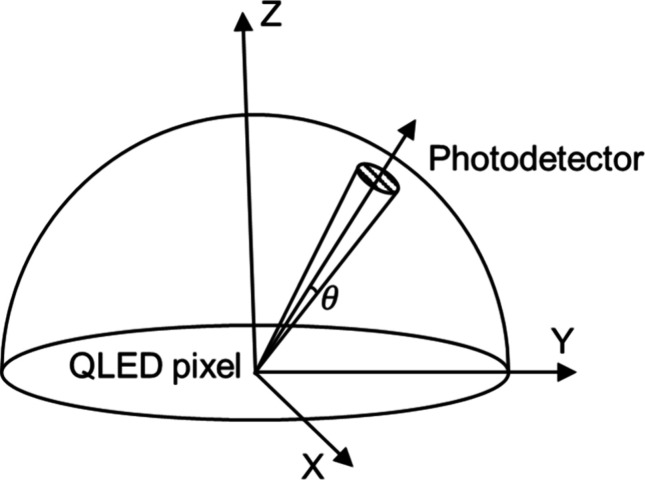
Schematic illustration of the geometric configuration used to calculate the solid angle subtended by the circular photodetector. The QLED pixel is approximated as a point source.

Luminance is the photometric quantity corresponding to radiance and is defined by spectrally weighting the emitted light with the photopic luminous efficiency function of the human eye.^95^ The photopic curve, *V*(λ), reflects the response of the human eye to different wavelengths of light. The photopic response function should be taken from the International Commission of Illumination (CIE) standard and converted to absolute units by multiplying the normalized function by *K*_m_ = 683 lm·W^–1^ (dataset can be found in ref 96). From the total hemispherical photon flux, luminance is calculated by applying the photopic weighting function, converting photon energy to optical power, and dividing by the device’s active area:

$$L=\frac{K_{m}{\cdot \Phi }_{\mathrm{phd}}\cdot \int V\left(\lambda \right)\cdot S\left(\lambda \right)\cdot \frac{hc}{\lambda }\;d\lambda }{A_{\mathrm{LED}}\cdot \int S\left(\lambda \right)\;d\lambda }$$

where *A*_LED_ (m^2^) is the active area of QLED defined by the overlapping of the anode and cathode.

To streamline the calculation of these key quantities, Python code that is available to the community (https://github.com/YIX421/LED-Data-Analysis) was developed. With this tool, users simply need to input the file pathway output from the data acquisition system described above. The code then processes data, outputs it into the same file, and generates corresponding plots. Thus, rapid evaluation of device performance is facilitated, allowing for easy comparison of different experimental variables.

## 7 Conclusion

Herein, a detailed QLED fabrication and characterization protocol using low-toxicity InP/ZnSe/ZnS QDs as a representative light-emitting active layer has been demonstrated. In addition, this work provides guidance on materials and device design strategies for improving QLED performance. To improve reproducibility, we first identify key sources of variability in QLED devices, particularly those arising from thin-film processing and interfacial properties. The processing methodology successfully fabricated devices with reproducible performance, yielding an average maximum EQE of ∼5.8% and a luminance of ∼8038 cd m^–2^. Independent validation by 3 researchers demonstrated the robustness and accessibility of the protocol. Furthermore, the QLED measurement system provides a cost-effective and automated solution for standardized measurement of devices. This protocol provides step-by-step instructions for researchers new to the field to establish the QLED platform with less effort, lowering the barrier to entering the field. Looking ahead, further improvements in QLED performance are expected through QD shell engineering, ligand chemistry, interfacial modification, layer thickness optimization, charge balance engineering, optical outcoupling structures, and tightly controlled fabrication conditions. We suggest that the community adopt a characterization checklist for QLED devices to improve transparency, similar to the checklist used for solar cells. Through establishing a standardized methodology for device fabrication and characterization, the design, development, and exploration of new device designs and materials that go beyond the state of the art will be facilitated.

## Supplementary Material





## Data Availability

Supporting code for the QLED data acquisition system and data analysis is available on GitHub at https://github.com/YIX421/LABVIEW-QLED-Measurement and https://github.com/YIX421/LED-Data-Analysis.

## Notes

Video S1, showing the step-by-step QLED fabrication instructions (MP4)
Video S2, demonstrating the QLED characterization workflow, including device measurement and automated data analysis procedures (MP4)
